# Self-care behaviors and associated factors among individuals with type 2 diabetes in Ghana: a systematic review

**DOI:** 10.1186/s12902-023-01508-x

**Published:** 2023-11-22

**Authors:** Richmond Opoku, Solomon Kwesi Ackon, Emmanuel Kumah, Charles Owusu-Aduomi Botchwey, Nana Esi Appiah, Shadrach Korsah, Michael Peprah

**Affiliations:** 1 Department of Health Administration and Education, University of Education, Winneba, Ghana; 2 Maxillofacial Surgery Unit, Korle-Bu Teaching Hospital, Accra, Ghana; 3 Mastercard Foundation Scholars Program, Kwame Nkrumah University of Science and Technology, Kumasi, Ghana

**Keywords:** Self-care behaviors, Adherence, Patient, Type 2 Diabetes, Systematic review, Ghana

## Abstract

**Background:**

Self-care remains an effective model for diabetes management and care in low-and-middle-income countries due to the limited resources available for the clinical management of the disease and its complications This study examined adherence to self-care behaviors and associated factors among people with type 2 diabetes in Ghana.

**Methods:**

PubMed, PsycINFO, Scopus, Web of Science, Embase and Google scholar were used to identify quantitative observational studies published between 1990 and September 30, 2023. Studies exclusive to persons with type 2 diabetes ≥ 18 years of age in a Ghanaian setting were included in this review. Findings of primary studies were analyzed using narrative synthesis.

**Results:**

Twelve studies, presenting data on a total of 2,671 persons with type 2 diabetes, were included. All the studies were published in the last decade (2015–2022) and a majority of them were from the Greater Accra Region. The mean number of days (per week) participants adhered to a self-care behavior were in the ranges of 3.9–4.4 for diet, 4.2–4.8 for physical activity, 0.5–2.2 for self-monitoring of blood glucose (SMBG), and 2.9–5.0 for foot care. Adherence rates for medication were in the range of 33.5–84.5%. Patient-related factors, sociodemographic/economic-related factors, condition-related factors, and healthcare system-related factors were associated with various self-care behaviors.

**Conclusion:**

Adherence to self-care behaviors among persons with type 2 diabetes in Ghana remains an ongoing challenge with significant variations in adherence among patients with different characteristics.

## Introduction

Diabetes continues to ravage healthcare systems in low-and-middle-income countries (LMICs), with an estimated 432.7 million people (over 80% of all diabetes cases globally) living with the disease in 2021 [1]. The International Diabetes Federation projects that this figure will rise to 665.5 million people (85% of all diabetes cases globally) by 2045 [1]. What this means is that the burden of diabetes in LMICs is likely to worsen if existing models of care are not strengthened to improve the management of diabetes cases in these countries. It is also important to note that diabetes mellitus is associated with acute and chronic health complications, and type 2 diabetes is the most common form of the disease, accounting for over 90% of all diabetes cases [2–4]. Unfortunately, healthcare systems in LMICs are not well developed to deal with the complications associated with diabetes, making the high burden of the condition in these parts of the world an issue of greater public health concern [5].

Self-care remains an effective model for diabetes management and care in LMICs due to the limited resources available for the clinical management of the disease and its complications [6, 7]. Patients who practice recommended self-care behaviors can enhance health outcomes and improve their quality of life [8, 9]. The self-care behaviors that exert a positive influence on diabetes outcomes include adherence to dietary recommendations, engaging in physical activity, medication adherence, self-monitoring of blood glucose (SMBG), and risk reduction measures such as foot care [10, 11]. Optimal adherence to these practices reduces susceptibility to diabetes complications and limits the burden of the disease on healthcare systems [12].

In Ghana, diabetes remains an emerging public health challenge, affecting 6.5% of the adult population [13]. Also, the control of cardio‑metabolic risk factors among persons with type 2 diabetes in Ghana is suboptimal [14], and this leads to massive costs for healthcare facilities managing the resulting complications [5]. On the back of these challenges, previous authors have highlighted the need to empower people with type 2 diabetes to take advantage of the many benefits associated with adherence to self-care behaviors [5, 15, 16]. This calls for a better understanding of the existing self-care practices among persons with type 2 diabetes in Ghana because interventions to empower these patients must be predicated on sound scientific evidence [6].

Although previous reviews have reported that adherence to diabetes self-care behaviors in LMICs are suboptimal [12, 17], no study has systematically synthesized information in the existing literature on self-care practices among persons with type 2 diabetes in Ghana. Consequently, there is no clear picture of the extent to which individuals with diabetes in Ghana adhere to self-care behaviors. This lack of information is a significant hurdle, as it prevents policy makers and clinicians from understanding whether diabetes self-care is optimal or not in this context. Without this crucial knowledge, it becomes challenging for Ghana to implement effective local measures to address the growing burden of type 2 diabetes on the healthcare system. This limitation not only affects Ghana’s ability to effectively manage diabetes, but also has broader implications for global efforts to improve diabetes management and care. A systematic review of studies from Ghana is indispensable in this regard – it will offer essential information to guide diabetes caregivers in their practice and interventions designed to empower patients to actively participate in the diabetes care and management process. Therefore, this study aims at synthesizing information in the existing literature to assess the extent of adherence to self-care behaviours among persons with type 2 diabetes in Ghana and to identify the associated factors.

## Methods

This systematic review was guided by the updated statement of the Preferred Reporting Items for Systematic Reviews and Meta-Analyses (PRISMA) [18].

### Literature search

A comprehensive literature search was conducted on PubMed, PsycINFO, Scopus, Web of Science, Embase and Google scholar for observational studies published between 1990 (when diabetes was recognized as a disease of public health concern in LMICs [12]) and September 30, 2023. The first search was conducted in September 2021 using a combination of keywords and medical subject headings (MeSH). Additional searches in March 2022, December 2022 and in October 2023 were subsequently conducted. The search string used in PubMed is as follows: “Self-Care“[Mesh]) OR (Self Care[Title/Abstract]) OR “Self-Management“[Mesh] OR “Medication Adherence“[Mesh]) OR (Medication Adherence[Title/Abstract]) OR (“Diet“[Mesh]) OR “Exercise“[Mesh]) OR (“Blood Glucose Self-Monitoring“[Mesh]) OR (Foot care[Title/Abstract]) AND (“Diabetes Mellitus, Type 2“[Mesh]) OR (Diabetes[Title/Abstract]) AND “Ghana“[Title/Abstract]. We also performed backward and forward hand-search using the reference lists and citations of included studies to identify relevant studies that might have been missed during the database search.

### Inclusion and exclusion criteria

A study qualified for inclusion if it had all the characteristics below:

i. **Population**: Individuals with type 2 diabetes ≥ 18 years of age.
ii. **Outcomes of interest**: prevalence of adherence to diabetes self-care behaviors or associated factors or both.
iii. **Context**: any setting in Ghana (community or health facility).
iv. **Study design**: quantitative observational study or a mixed-methods observational study with quantitative findings on any of the outcomes of interest.

Studies not meeting any of the criteria for inclusion were excluded. Studies that were not exclusive to persons with type 2 diabetes in Ghana were excluded. Also, studies investigating the effectiveness of interventions among individuals with type 2 diabetes were not included. Finally, qualitative, longitudinal, and secondary studies were excluded from this review.

### Study selection and data extraction

Titles of the records identified were initially screened by three reviewers (R.O, SKA, EK) and all titles that appeared to meet the inclusion criteria were exported into a Microsoft Excel file for further assessments. Four reviewers (RO, SKA, NEA, EK) screened the abstracts of the included titles and subsequently assessed the full-text articles against the inclusion criteria. All disagreements during this stage were resolved through discussion and consensus building. Data extraction was done by three reviewers (RO, SKA, EK) independently using a Microsoft Excel template developed to collect data on the following study characteristics: author and year of publication, study title, data collection period, study location, study methods (design, sampling, and sample size), age of participants, data collection instrument, and findings on the outcomes of interest. The extracted data was then checked by the remaining reviewers for accuracy (ensuring that the data was free from errors and inaccuracies), and completeness (ensuring that no important information had been missed during the extraction process).

### Quality assessment

The methodological quality of the included studies was assessed using the Mixed Methods Appraisal Tool (MMAT) Version 2018 [19]. The MMAT is a quality appraisal tool developed to evaluate the methodological quality of empirical studies. The tool can appraise five different categories of study designs: qualitative, randomized controlled trial, non-randomized, quantitative descriptive and mixed methods studies [19]. The quantitative descriptive category was chosen for this study as all of the included studies were cross-sectional. This category has five methodological quality criteria, with three response options: “Yes” (meaning the criterion is met), “No” (meaning the criterion is not met), and “Can’t tell” (indicating there is no enough information in the paper to judge if the criterion is met or not) (Table 1).

**Table 1:** Quality assessment tool used to evaluate the methodological quality of the included studies

Methodological quality criteria	Responses		
	Yes	No	Can’t tell
Is the sampling strategy relevant to address the research question?			
Is the sample representative of the target population?			
Are the measures appropriate?			
Is the risk of non-response bias low?			
Is the statistical analysis appropriate to answer the research question?			

Adopted from Hong et al. [19]

Three reviewers (RO, EK, COAB) jointly conducted the appraisal. Studies that received a “Yes” response to ≤ 2, 3 and ≥ 4 of the questions were deemed to be low quality, moderate quality, and high quality respectively. All disagreements were resolved through discussion and consensus building.

### Data analysis and synthesis

A narrative synthesis was conducted in this review since the included studies were not sufficiently similar to allow statistical meta-analysis [12]. The analysis and synthesis were conducted following recommendations from the Guidance of the Conduct of Narrative Synthesis in Systematic Reviews [20]. Specifically, tabulation and grouping strategies were used to organize the findings of the review under the various self-care behaviors and the WHO’s five dimensions on adherence to therapies among patients (patient-related factors, sociodemographic/economic related factors, condition-related factors, therapy-related factors, and healthcare system-related factors) [21].

## Results

### Study selection

**Fig. 1:**
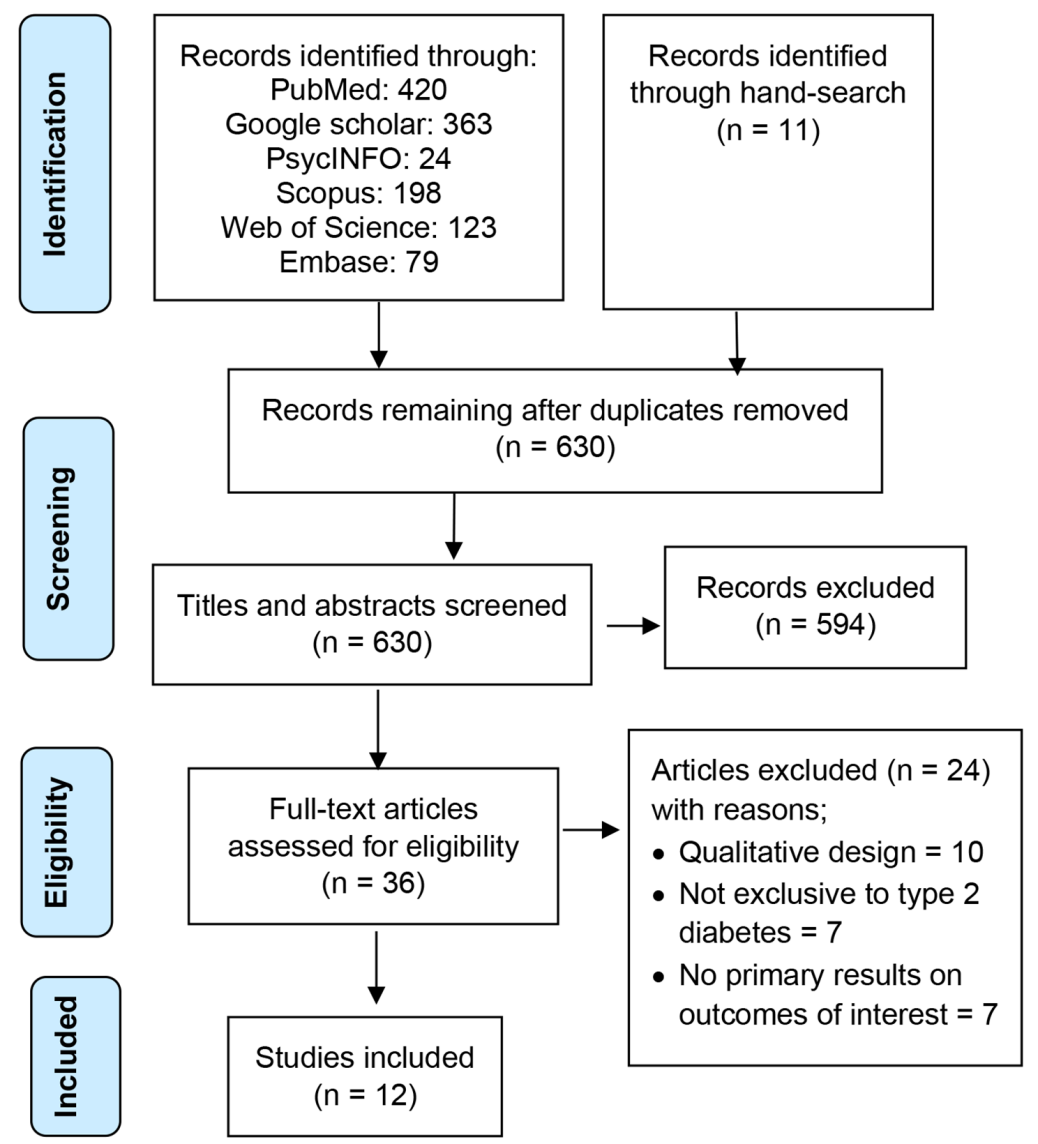
PRISMA flow chart showing the study selection process and results

The results of the study selection at the various phases of this review have been presented in Fig. 1. Our literature search yielded a combined total of 1,218 titles from the database and hand searches. After duplicates were removed, 630 titles were retained for further screening. After title and abstract screening, 594 records that were not relevant to the objectives of the review were discarded leaving 36 articles for full-text assessment. A total of 12 full-text articles met the inclusion criteria and the remainder of the 24 articles were excluded for the following reasons: was a qualitative study (n = 10), was not exclusive to persons with type 2 diabetes (n = 7), and did not present primary results on any outcome of interest (n = 7).

### Characteristics of included studies

The characteristics of the included studies have been presented in Table 2. All the studies were published within the last decade (2015–2022) and were cross-sectional studies. Half of the studies that reported their sampling strategy used a probability sampling technique and the remaining half used a non-probability sampling technique. The studies had a combined sample size of 2,671 persons with type 2 diabetes, given that two of the studies [11, 22] were considered as one since they reported data on the same sample but for different outcomes of interest. The included studies were conducted in seven (43.8%) of the sixteen regions in Ghana (Brong Ahafo Region was taken as representing three distinct regions). Nearly half of the studies (45.5%) reported data from the Greater Accra Region. Seven (58.3%) of the studies assessed a single self-care behavior and only two (16.7%) assessed all five self-care behaviors of interest in this review. The included studies used different measuring tools to collect data (Table 2).

**Table 2:** Characteristics of included studies

Study	Year	Study methods	Region	Behavior assessed	Measurement tool
Afaya et al. [23]	2020	*Design*: Descriptive cross-sectional *Sampling strategy*: Consecutive *Sample size*: 330	Northern Region	Diet, exercise, SMBG, foot care, medication	Summary of diabetes self-care activities (SDSCA) & Medication adherence questionnaire (MAQ)
Amankwah-poku et al. [24]	2020	*Design*: Descriptive cross-sectional *Sampling strategy*: Convenience *Sample size*: 162	Greater Accra Region	Diet, exercise, medication	Summary of diabetes self − care activity scale (DSCA)
Bruce et al. [25]	2015	*Design*: Descriptive cross-sectional *Sampling strategy*: Systematic random *Sample size*: 200	Greater Accra Region	Medication	Morisky medication adherence scale (MMAS)
Doglikuu et al. [11]	2021	Design: Descriptive cross-sectional *Sampling strategy*: Systematic random *Sample size*: 530	Brong Ahafo Region*	Diet	Perceived dietary adherence questionnaire (PDAQ)
Doglikuu et al. [22]	2021	*Design*: Descriptive cross-sectional *Sampling strategy*: Systematic random *Sample size*: 530	Brong Ahafo Region*	Exercise	WHO physical activity-short form questionnaires
Kretchy et al. [ [16]	2020	*Design*: Descriptive cross-sectional *Sampling strategy*: simple random *Sample size*: 188	Greater Accra Region	Medication	Medication adherence report scale (MARS)
Kugbey et al. [26]	2017	*Design*: a cross-sectional survey *Sampling strategy*: Convenience *Sample size*: 160	Greater Accra Region	Diet, exercise, SMBG, foot care, Medication	Self-care practices questionnaire
Mogre et al. [27]	2017	*Design*: a cross-sectional survey *Sampling strategy*: Not reported *Sample size*: 187	Northern Region	Diet, exercise, SMBG, foot care	Summary of diabetes self-care activities (SDSCA)
Osei-yeboah et al. [8]	2018	*Design*: Descriptive cross-sectional *Sampling strategy*: Purposive *Sample size*: 150	Volta Region	Medication	Morisky, Green, and Levine Adherence Scale (MGLS)
Owiredua et al. [28]	2018	*Design*: cross-sectional exploratory *Sampling strategy*: Purposive & convenience *Sample size*: 196	Greater Accra Region	Medication	Medication adherence report scale (MARS-5)
Sefah et al. [29]	2020	*Design*: Descriptive cross-sectional *Sampling strategy*: Random *Sample size*: 400	Volta Region	Medication	Morisky medication adherence scale (MMAS-8)
Nketia [30]	2022	*Design*: Descriptive cross-sectional *Sampling strategy*: Systematic random *Sample size*: 168	Savannah Region	Medication, diet, physical activity	Medication Adherence Rating Scale (MARS) & Exercise Adherence Rating Scale (EARS)

*Now exists as three distinct regions (i.e., Ahafo Region, Bono Region, and Bono-East Region)

### Quality assessment

The results of the quality assessment presented in Table 3 show that all the included studies had moderate to high quality, with a majority of them (66.7%) rated as high quality. Common limitations included the use of non-probability sampling strategies without justification [8, 23–26], and limited description of sampling methods [13, 24, 26, 29].

**Table 3:** Quality assessment of the included studies

Study	Overall Rating
Afaya et al. [23]	High
Amankwah-Poku et al. [24];	High
Bruce et al. [25]	Moderate
Doglikuu et al. [11]	High
Doglikuu et al. [22]	High
Kretchy et al. [6]	High
Kugbey et al. [26]	Moderate
Mogre et al. [27]	Moderate
Osei-Yeboah et al. [8]	High
Owiredua et al. [28]	High
Sefah et al. [29]	High
Nketia [30]	Moderate

### Adherence to self-care behaviors among people with type 2 diabetes in Ghana

The results on the prevalence of adherence to self-care behaviors have been presented in Table 4. These have been grouped under the various self-care behaviors.

#### Diet

Four studies reported primary results on the extent of adherence to dietary recommendations [22, 23, 27, 30]. Two studies found that on average, individuals with type 2 diabetes adhered to recommended dietary practices about four days a week [23, 27]. Another study reported a mean total adherence to dietary recommendations of 32.6. Mogre et al. [27] further reported that the proportion of patients adhering to dietary recommendations daily was 2.7% of patients [27].

#### Physical activity

Three studies reported data on the extent of adherence to physical activity recommendations among individuals with type 2 diabetes [23, 27, 30]. One study reported that patients engaged in physical activity for approximately four days [23]. Another study found approximately five days of physical activity among participants [27]. Further, Mogre et al. [27] reported the daily adherence to recommendations on physical activity to be 21.4% among participants. The overall adherence to physical activity reported by Nketia [30] was 41.8%.

#### Medication

Six of the included studies reported data on the extent of adherence to medication among persons with type 2 diabetes [8, 16, 23, 25, 29, 30]. These studies reported varying rates of good adherence to medication among the study participants in the range of 33.5–84.5%. The highest optimal proportion of adherence (84.5%) was reported by Afaya et al. [23] and the lowest (33.5%) by Kretchy et al. [16]. Three studies found optimal adherence to medication to be prevalent in more than half of the participants [8, 23, 30].

#### Self-monitoring of blood glucose (SMBG)

Two studies reported on the extent of adherence to SMBG among persons with type 2 diabetes in Ghana [23, 27]. One study found that participants performed SMBG barely a day in the last seven days [21]. The other study reported that persons with type 2 diabetes performed SMBG on average, approximately two days a week and that only one (0.5%) of all patients studied performed SMBG daily in the past one week [27].

#### Foot care

Two studies reported data on the extent of adherence to foot care recommendations [23, 27]. One study reported that the study prticipants practiced foot care on average five days a week [23]. The other study found that individuals with type 2 diabetes practiced foot care on average, approximately three days a week and that 9.6% of them practiced it daily [27].

**Table 4:** Adherence to self-care behaviors among type 2 diabetes patients in Ghana

Study	Self-care behavior	Reported measure	Adherence level
Afaya et al. [23]	Diet	Mean (SD) number of days participants adhered to total dietary recommendations during the last 7 days.	3.9 (1.0)
Doglikuu et al. [11]		Mean (SD) of total adherence to recommended dietary guidelines.	32.6 (9.6)
Mogre et al. [27]		Mean (SD) number of days participants adhered to total dietary recommendations during the last 7 days.	4.40 (1.52)
Mogre et al. [27]		Number (%) of patients who adhered to total dietary recommendations daily in the last 7 days.	5 (2.7%)
Nketia [2022]		Prevalence of optimal adherence to dietary recommendations.	56.0%
Afaya et al. [23]	Physical activity	Mean (SD) number of days participants engaged in physical activity (including exercise sessions) during the last 7 days.	4.2 (2.3)
Mogre et al. [27]		Mean (SD) number of days participants engaged in physical activity (including exercise sessions) during the last 7 days.	4.78 (2.09)
Mogre et al. [27]		Number (%) of patients who engaged in physical activity (including exercise sessions) daily in the last 7 days.	40 (21.4%)
Nketia [2022]		Prevalence of optimal adherence to physical activity.	41.8%
Afaya et al. [23]	Medication	Prevalence of optimal adherence to the medication regimen.	84.50%
Bruce et al. [25]		Prevalence of optimal adherence to the medication regimen.	38.50%
Kretchy et al. [16]		Prevalence of optimal adherence to the medication regimen.	33.51%
Osei-Yeboah et al. [8]		Prevalence of optimal adherence to the medication regimen.	60.67%
Sefah et al. [29]		Prevalence of optimal adherence to the medication regimen.	47.7%
Nketia [2022]		Prevalence of optimal adherence to the medication regimen.	51.8%
Afaya et al. [23]	SMBG	Mean (SD) number of days participants self-monitored blood glucose in the last 7 days.	0.5 (1.3)
Mogre et al. [27]		Mean (SD) number of days participants self-monitored blood glucose in the last 7 days.	2.2 (0.65)
Mogre et al. [27]		Number (%) of patients who performed SMBG daily in the last 7 days.	1 (0.5%)
AAfaya et al. [23]	Foot care	Mean (SD) number of days participants practiced foot care during the last 7 days.	5.0 (1.3)
Mogre et al. [27]		Mean (SD) number of days participants practiced foot care during the last 7 days.	2.9 (2.16)
Mogre et al. [27]		Number (%) of patients who practiced foot care daily in the last 7 days.	18 (9.6%)

### Factors associated with adherence to type 2 Diabetes self-care behaviors in Ghana

The results on the factors associated with self-care practices have been presented in Table 5. These have been grouped under broad themes according to the WHO dimensions [21]. No factor was found in the literature under the therapy-related factors of the WHO dimensions.

#### Patient-related factors

One study found that participants with high knowledge of diabetes were more likely to adhere to overall self-care practices as well as dietary, SMBG, and foot care recommendations [26]. Two studies reported that people with type 2 diabetes who expressed negative illness representations were less likely to adhere to overall self-care practices, diet, exercise, and medication recommendations [26, 28]. However, patients with high perceptions of personal control were more likely to adhere to medication regimens [28].

#### Sociodemographic/economic-related factors

Two studies reported that male individuals with type 2 diabetes were more likely to perform SMBG compared to females [23, 27]. Older patients were more likely to adhere to medication [23], but less likely to adhere to dietary recommendations [22]. High levels of education were associated with increased adherence to dietary, exercise, and foot care recommendations [27]. However, a contrary finding showed that high levels of education were associated with poor adherence to dietary recommendations [23]. Three studies reported that high levels of education increased the likelihood of medication adherence [23, 25, 29]. Being married and having high socioeconomic status were both associated with increased levels of adherence to dietary recommendations [11]. Also, having high socioeconomic status and social support increased the likelihood of adherence to cardiovascular fitness recommendations among people with type 2 diabetes [22]. However, social support negatively predicted adherence to diet, exercise, and medication adherence [9, 22]. One study reported that owning a personal glucometer was associated with an increased frequency of SMBG among individuals with type 2 diabetes [23].

#### Condition-related factors

It has been reported that high diabetes distress reduces adherence to diet, exercise, and medication recommendations among people with type 2 diabetes [16, 24]. High diabetes-related emotional distress also reduces the likelihood of exercising among individuals with type 2 diabetes [24].

#### Health-care system-related factors

Strangely, one study reported that people with type 2 diabetes who received all their prescribed medications at the hospital pharmacy were less likely to take them compared to those who did not receive one or two medications prescribed to them [29].

**Table 5:** Factors associated with self-care behaviors among type 2 diabetes patients in Ghana

Factor	Reported results
1. Patient-related	
Diabetes knowledge	High knowledge of diabetes is associated with increased adherence to overall self-care practices, diet, SMBG, and foot-care recommendations [26].
Illness representation	Negative illness representations (i.e., belief that diabetes has serious negative impacts on a patient’s life and exhibiting negative emotions) were associated with poor adherence to overall self-care practices, diet, exercise [25], and medication recommendations [28].
	Perception of personal control (i.e., belief in personal ability to act to improve health) was positively associated with adherence to medication recommendations [28].
**2. Sociodemographic/economic-related**	
Gender	Male participants had a higher frequency of SMBG compared to female participants [23, 27].
Age	Elderly participants (≥ 70 years) were more likely to adhere to medication recommendations compared to those below 50 years [23].
	Age was negatively associated with adherence to dietary recommendations [11].
Education	High levels of education were associated with increased adherence to diet, exercise, and foot care recommendations [27].
	The number of years in school was negatively associated with adherence to dietary recommendations [23].
	High levels of education were associated with increased adherence to medication recommendations [23, 25, 29].
Marital status	Being married was associated with increased adherence to diet [1].
Socioeconomic status	Socioeconomic status was positively associated with adherence to diet and cardiovascular fitness recommendations [11, 22].
Social support	Social support was negatively associated with adherence to diet, exercise, and medication recommendations [11, 24].
	Social support was positively associated with adherence to cardiovascular fitness recommendations [11].
Ownership of glucometer	Owning a glucometer was associated with an increased frequency of SMBG [23].
**3. Condition-related**	
Diabetes distress	Diabetes distress is negatively associated with adherence to diet, exercise, and medication recommendations [16, 24].
Diabetes-related emotional distress	Diabetes-related emotional distress is negatively associated with adherence to an exercise regimen [24].
**4. Health-care system-related**	
Drug availability at the hospital	The availability of anti-diabetic drugs in the hospital pharmacy was negatively associated with adherence to medication adherence [29].

## Discussion

Diabetes remains a global public health challenge, having its greatest burden in the past decade among LMICs [1]. Until this review, the extent of adherence to self-care behaviors among individuals with type 2 diabetes in Ghana and the associated factors were poorly understood. This review synthesized the available literature in Ghana to bring clarity to this neglected aspect of diabetes research in the country. The review included 12 observational studies, all of which were published in the last decade (2015–2022). This finding reflects the emerging nature of diabetes in Ghana and most LMICs. Until the 1990s diabetes was not recognized as an issue of public health concern in most developing countries, including Ghana [12]. Thus, it is not surprising that all the included studies were published quite recently. The majority of the included studies were conducted in the Greater Accra Region. Since more people living with diabetes are in urban areas compared to rural areas [1], this finding was expected as the Greater Accra Region is the most urbanized in the country, with 91.7% of its population residing in urban areas [31].

We observed poor level of adherence to dietary recommendations among people with type 2 diabetes in this review. This finding, while illuminating, diverges from the outcomes reported in earlier studies, where adherence rates were identified as moderate, ranging from 33% to as high as 91% in sub-Saharan Africa [17] and other LMICs [27]. The poor level of adherence to dietary recommendations observed in the present study might be explained by the difficulty most people with diabetes face in abandoning long-held taste for traditional Ghanaian foods and the inability to resist the temptation to defy dietary recommendations at social functions such as funerals and weddings which are very common in many Ghanaian communities [32]. Previous evidence from elsewhere shows that it is difficult to adopt new eating habits at a later age [33], and the influence of social pressure and the cultural meaning of food on adherence to dietary recommendations cannot be overemphasized [34, 35].

On the contrary, the adherence to regular exercise demonstrated in this study was very commendable, with participants averaging an impressive four to five days of exercise per week (23,27). This level of dedication surpasses what has been previously observed in other populations, such as Filipino-Americans [36] and African Americans [37] with type 2 diabetes, but parallels the findings of a study conducted in Ethiopia [38]. The high level of adherence found in this review might be attributed to the predominantly urban settings of the studies, where fitness centers are more accessible. Despite the encouraging weekly exercise, it is essential to highlight that only a minority of participants managed to adhere to daily exercise recommendations [27]. A potential explanation for this could be a lack of support for exercise [39]. This lack of support may encompass various facets, including insufficient guidance from healthcare providers, and a dearth of social encouragement and motivation.

Medication adherence, a critical aspect of self-care behavior, has garnered significant attention in the literature. This review has revealed a wide variation in medication adherence rates, and this variation may be attributed to several factors. Previous studies in Ghana have reported existing disparities in income levels, limited access to comprehensive healthcare services due to a lack of comprehensive insurance coverage, and challenges related to the availability of medicines [40, 41]. These factors might have contributed to the observed differences in medication adherence rates among the included studies. Addressing these multifaceted challenges is crucial for improving medication adherence among individuals with type 2 diabetes and, subsequently, the overall health outcomes of the people in Ghana.

The present review highlights a concerning trend among individuals with type 2 diabetes in terms of their adherence to recommended blood glucose monitoring practices. It is worth noting that this observation is consistent with the findings of a similar review conducted in sub-Saharan Africa [7]. The overarching message gleaned from these findings is the glaringly low adherence to SMBG as a crucial aspect of diabetes management. Consequently, evidence-based interventions are warranted to address this issue and enhance the overall quality of care and management of type 2 diabetes in Ghana. These interventions should encompass a multifaceted approach that goes beyond conventional behavior change strategies [42]. While these educational interventions (behavior change strategies) are valuable, they may not be sufficient on their own to drive meaningful change in SMBG practices.

In relation to the patient-related factors, we observed that individuals with a higher level of diabetes knowledge were more likely to adhere to self-car practices, including dietary recommendations, SMBG and foot care [26]. This finding is consistent with the results of previous studies from other countries that found diabetes knowledge to have a positive influence on self-care practices [43]. As suggested by Kugbey et al. [26], diabetes caregivers should identify and consider the individual educational needs of diabetes patients to enhance the uptake of self-care practices among people with type 2 diabetes. The review also found that negative illness representations were associated with poor adherence to self-care practices [2, 28], while positive perceptions of personal control had a positive association with medication adherence [28]. When patients have threatening views about their illness, they are likely to adopt fatalistic views on their condition [26] and get emotionally distressed [16]. In turn, they adopt fewer self-care behaviors, increasing their risk of developing diabetic complications. The implication of this finding is the need for a holistic approach to diabetes care and management in Ghana, where a patient’s psychological and emotional reaction to the disease is considered very essential to the management course. Specifically, diabetes caregivers must understand patients’ representations of diabetes to plan interventions that aim at empowering them to not give up on themselves, but to take control of their condition.

The review also found among the sociodemographic/economic-related factors that men were more likely to perform SMBG compared to women [23, 27]. This could be explained by the fact that women are more likely to engage in self-blame and be taken over easily by their emotions while men are determined to perform the more technical aspects of their care [27]. Caregivers should take cognizance of these gender differences and understand the unique needs of women during counseling. We also found that older patients were more likely to adhere to medication [23] but less likely to have appropriate dietary practices [11]. The report on the relationship between age and medication adherence is consistent with the results of a previous review of studies from LMICs [2]. Younger people are more likely to be actively employed, sometimes with busy schedules, and much healthier and as such may be tempted not to adhere to medications. At the same time, older people are usually retired with very inadequate social security to cater to their dietary needs.

We found among the condition-related factors that both diabetes distress and diabetes-related emotional distress were negatively associated with adherence to self-care behaviors among persons with type 2 diabetes in Ghana [16, 24]. Diabetes caregivers must take due notice of these relationships to incorporate psychological interventions into the care and management of diabetes patients. In another dimension (health-care system-related factors*)*, and more strangely, one study found that receiving all prescribed medicines at the hospital pharmacy was associated with decreased adherence to medication [29]. The authors failed to provide any explanation for this rather inexplicable finding, and we also did not find any empirical literature reporting similar findings. Further research is therefore needed to explore into this relationship.

### Limitations

This systematic review, being among the few to provide a clearer understanding of the practice of self-care among people with type 2 diabetes in Ghana, was conducted on the back of a comprehensive literature search. The use of MeSH terms, hand-search, and the four searches at different time intervals increased the likelihood of identifying all relevant studies for this review. Also, all included studies were found to have moderate to high quality, even though study quality was not used as a criterion for exclusion. This implies that this review included the best available evidence on the practice of self-care behaviors in Ghana. However, this review has some limitations that need to be discussed. A first limitation relates to the rigorous inclusion and exclusion criteria we adopted. For instance, we restricted the search strategy to only English language publications. This may have resulted in relevant information in studies published in other languages being excluded from our analysis. Second, the inclusion of only 12 studies in the review is an indication that the conclusions drawn are based on limited data. Third, we could not provide statistically pooled estimates of the extent of adherence to various self-care behaviors due to the nature of heterogeneity in the included studies. However, following appropriate guidelines to perform a narrative synthesis gave us the best opportunity to make meaning from the existing literature. Therefore, notwithstanding these limitations, this review provides useful insights that will improve type 2 diabetes care and management in Ghana and similar settings where such reviews are nonexistent.

## Conclusion

This review has revealed that adherence to self-care behaviors among people with type 2 diabetes in Ghana is poor. Levels of non-adherence to SMBG and foot care are particularly troubling and require urgent efforts to remedy the situation. Several factors within the WHO framework of factors associated with non-adherence to therapy were found to influence the practice of self-care among individuals with type 2 diabetes in Ghana. These included patient-related factors, sociodemographic/economic-related factors, condition-related factors, and healthcare system-related factors. Interventions, as well as further research, are needed to improve the practice of self-care behaviors among people with type 2 diabetes in Ghana.

## Data Availability

No datasets were generated or analyzed during the current study.

## Abbreviations

LMC: Low–and–Middle–Income Country
MMAT: Mixed Methods Appraisal Tool
PRISMA: The Preferred Reporting Items for Systematic Reviews and Meta–Analyses
SMBG: Self–Monitoring of Blood Glucose
WHO: World Health Organization
